# Mora: abundance aware metagenomic read re-assignment for disentangling similar strains

**DOI:** 10.1186/s12859-024-05768-9

**Published:** 2024-04-23

**Authors:** Andrew Zheng, Jim Shaw, Yun William Yu

**Affiliations:** 1 Mathematics, University of Toronto, 27 King’s College Circle, Toronto, Ontario M3R 0A3 Canada; 2 Computer and Mathematical Sciences, University of Toronto at Scarborough, 1265 Military Trail, Toronto, Ontario M1C 1A4 Canada; 3 Ray and Stephanie Lane Computational Biology Department, Carnegie Mellon University, 5000 Forbes Ave, Pittsburgh, Pennsylvania 15213 USA

**Keywords:** Metagenomics, Read re-assignment, Abundance quantification

## Abstract

**Background:**

Taxonomic classification of reads obtained by metagenomic sequencing is often a first step for understanding a microbial community, but correctly assigning sequencing reads to the strain or sub-species level has remained a challenging computational problem.

**Results:**

We introduce Mora, a MetagenOmic read Re-Assignment algorithm capable of assigning short and long metagenomic reads with high precision, even at the strain level. Mora is able to accurately re-assign reads by first estimating abundances through an expectation-maximization algorithm and then utilizing abundance information to re-assign query reads. The key idea behind Mora is to maximize read re-assignment qualities *while simultaneously* minimizing the difference from estimated abundance levels, allowing Mora to avoid over assigning reads to the same genomes. On simulated diverse reads, this allows Mora to achieve F1 scores comparable to other algorithms while having less runtime. However, Mora significantly outshines other algorithms on very similar reads. We show that the high penalty of over assigning reads to a common reference genome allows Mora to accurately infer correct strains for real data in the form of *E. coli* reads.

**Conclusions:**

Mora is a fast and accurate read re-assignment algorithm that is modularized, allowing it to be incorporated into general metagenomics and genomics workflows. It is freely available at https://github.com/AfZheng126/MORA.

**Supplementary Information:**

The online version contains supplementary material available at 10.1186/s12859-024-05768-9.

## Background

When analyzing microbial communities through metagenomic sequencing, a fundamental task is to determine which reference genome a specific sequencing read originates from [1]. This gives information about microbial composition and allows for mapping-based analysis of genetic variation. A common first step in a processing pipeline is to use a fast taxonomic read classifier such as Kraken2 [2], CLARK [3], Centrifuge [4] or others. While such methods are extremely fast, they are not sensitive enough to assign reads to the strain level. Since strain-level resolution has important functional implications [5–7], sensitive methods that are able to resolve reads at the strain level are needed.

To resolve reads at the level of strains, a naive approach would be to more sensitively align the read to a set of candidate reference genomes using a read aligner such as Bowtie2 [8], Minimap2 [9], or CORA [10] (no relation to Mora) and then take the best reference genome as the correct assignment. However, strain-level reference genomes share large regions of similarity, so many ambiguous mappings are inevitable. To overcome this limitation, one can statistically calculate abundance information of the candidate set of reference strains and only use references that have high enough abundances [11–14]. Afterward, one can re-assign reads to the “correct” references, i.e. reference strains that seem to be abundant in the metagenomic sample. This approach has been used to guide short-read strain-level disambiguation of skin metagenomes [15]. It is currently used in long-read taxonomic profiling with least common ancestor approaches to better quantify profiling ambiguity [16].

However, re-assigning reads to the correct references while maintaining abundance estimates is non-trivial. For example, if one were to assign a multi-mapped read to the most abundant reference strain with a putative mapping, all reads coming from a region of similarity between two strains will be mapped to only one strain—in this case the most abundant one. This is not an accurate assignment of the reads and will skew the abundance. Importantly, not all algorithms do abundance estimation *and* read assignment; some algorithms calculate only abundances and do not output re-assigned reads [17–19].

In this paper, we present Mora, a tool that allows for sensitive yet efficient metagenomic read re-assignment and abundance calculation at the strain level for both long and short reads. Given an alignment in SAM or BAM format and a set of reference strains, Mora calculates the abundance of each reference strain present in the sample and re-assigns the reads to the correct reference strain in a way such that abundance estimates are preserved. We rigorously formulate this problem as an optimization problem and give provable guarantees on our heuristic algorithm. We show that Mora is more effective than Pathoscope2 [12], a state-of-the-art read re-assigner, at disambiguating similar strains present in a sample on simulated data for short reads while being an order of magnitude faster. Furthermore, we show that Mora has similar F1 scores compared to Pathoscope2, Kraken2, and Clark while taking less time and RAM on simulated long read data. We then verify our results on real data as well.

## Results and discussion

### Pipeline of Mora

**Fig. 1:**
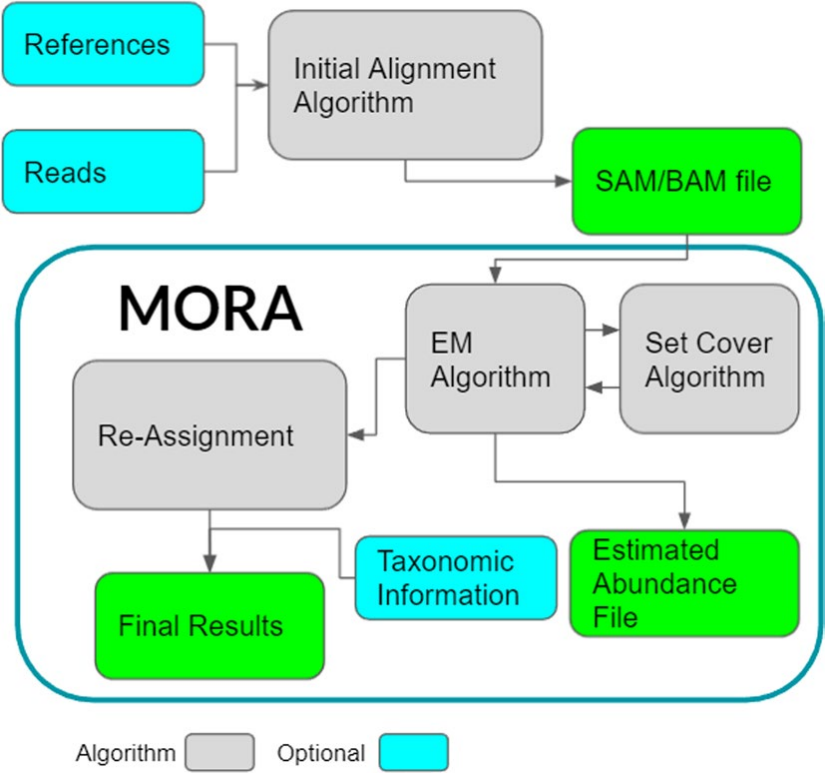
The pipeline for Mora to output re-assignments from query and reference FASTA files. Mora’s processing steps are enclosed by the dark green rectangle. The abundance estimation step includes the Expectation Maximization (EM) algorithm and a set cover algorithm to filter out spuriously abundant genomes [19]. The re-assignment step uses the estimated abundance from the previous step to re-assign reads

**Fig. 2:**
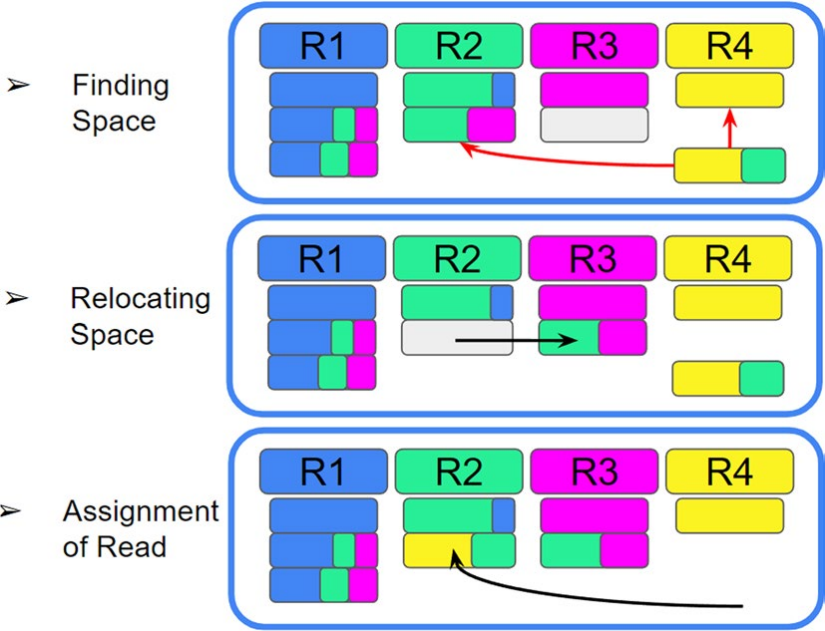
An example of Mora’s approach to read re-assignment. The exact algorithm is outlined in “Re-assignment of reads” section. R1–R4 are four reference genomes labelled with colours, and the 8 reads shown have colour content proportional to the mapping scores with respect to each coloured reference genome. Grey boxes are not reads, but available read assignments based on Mora’s estimated abundances. Step 1: As shown in the red arrows, Mora cannot assign the last read due to the lack of space in R2 and R4 from to the abundance constraint Step 2: shown in the black arrow, we move a more ambiguously assigned read in R2 to R3 instead, opening space in R2. Step 3: We move the un-assigned read to R2, where there is now space

Mora’s pipeline consists of two main steps (Fig. 1): abundance estimation and read re-assignment. Like other metagenomic abundance estimators [19, 20], it first utilizes a standard generative probabilistic model on input mappings and performs inference using the expectation maximization (EM) algorithm, augmented with a set cover algorithm to filter out spuriously abundant genomes [19]. Mora’s novelty comes from the subsequent step, where we re-assign reads in a manner *dependent on the calculated abundances*. Though optimization with constraints has been used in abundance estimation before [21], our constraints and our specific reassignment procedure are novel. Mora models the problem of maximizing correct read assignments while minimizing the difference between predicted abundance levels and final abundance levels as a non-linear minimization problem (“Re-assignment of reads” section and Fig. 2). Although we use a greedy heuristic, we prove that the heuristic is guaranteed to improve the minimization score. Finally, Mora outputs a (re-)assignment of each read to a reference genome that is consistent with the calculated abundances.

### Benchmarking details

We benchmarked Mora using simulated short and long reads against Pathoscope2 [12], Kraken2 [2], and Clark [3]. Pathoscope2 can identify strains, compute abundance levels, perform re-assignments, and output informative result summaries. It has seen usage in numerous studies [22–25], and its algorithm has also been incorporated into other taxonomic classification pipelines [16]. Kraken2 and Clark are two taxonomic classifiers that classify reads by assigning them a taxonomic ID. In the case of Kraken2, it may classify reads to the least common ancestor of the possible taxons to reduce the chance of false assignments. For Clark, the taxonomic IDs can be manipulated for strain-level assignments. This was also attempted for Kraken2, but errors were encountered when trying to build a custom database. Hence, Kraken2 was unable to work at the strain level. When aligning simulated reads to the bacteria RefSeq database, Clark was unable to correctly build the current 2024 January version from NCBI. A 2019 version of the RefSeq bacteria database was attempted as well, but in this case it could not even finish building the database. Hence, CLARK’s standard 2024 database version was used even though it gave very low scores (see “Large-scale simulated short read re-assignment at the species and genus level” section). However, Clark still was able to build smaller sized custom databases (REF-1).

All algorithms except Pathoscope2 were run using their default parameters, which would not run due to the high number of strains. Instead, certain parts in Pathoscope2’s code had to be augmented to allow it run correctly. Its initial library construction code no longer works and must be replaced with code from MetaScope [26]. The output step also had to be fixed to allow for large number of reads. These changes did not affect how Pathoscope2 re-assigns reads, so the only change should be being able to run on large datasets and a decrease in runtime. We now denote the augmented Pathoscope2 as AugPatho2, and the augmented code can be found in same repository as the Additional file 1: Appendix files. Given these caveats, Pathoscope2 appears to be the current practical state-of-the-art.

We also considered MetaMaps [14] and Sigma [13], two other re-assignment tools, but they were not included in this comparison as the software are no longer actively maintained and parts of both software are no longer functional; other studies also had issues with these two programs [17, 27].

For initial aligners, we mainly use Pufferfish/Puffaligner [28] and Bowtie2 for short reads. Pufferfish is faster and more memory efficient compared to Bowtie2, but at the cost of being less sensitive. These were chosen to see how well Mora and AugPatho2 performs based on very sensitive aligner compared to very fast aligners. Minimap2 is used for long reads due to its popularity and ease of use.

F1 score, sensitivity, and precision are used to evaluate the accuracy of the final read re-assignment at three different taxonomic ranks: strain, species, and genus (see “Evaluation metrics” section). For the purpose of this paper, two DNA sequences are of the same species/genus if their NCBI taxID corresponds to the same species/genus name. Two DNA sequences are of the same strain if their accession number is the same.

### Re-assignment of reads to similar strains

**Fig. 3:**
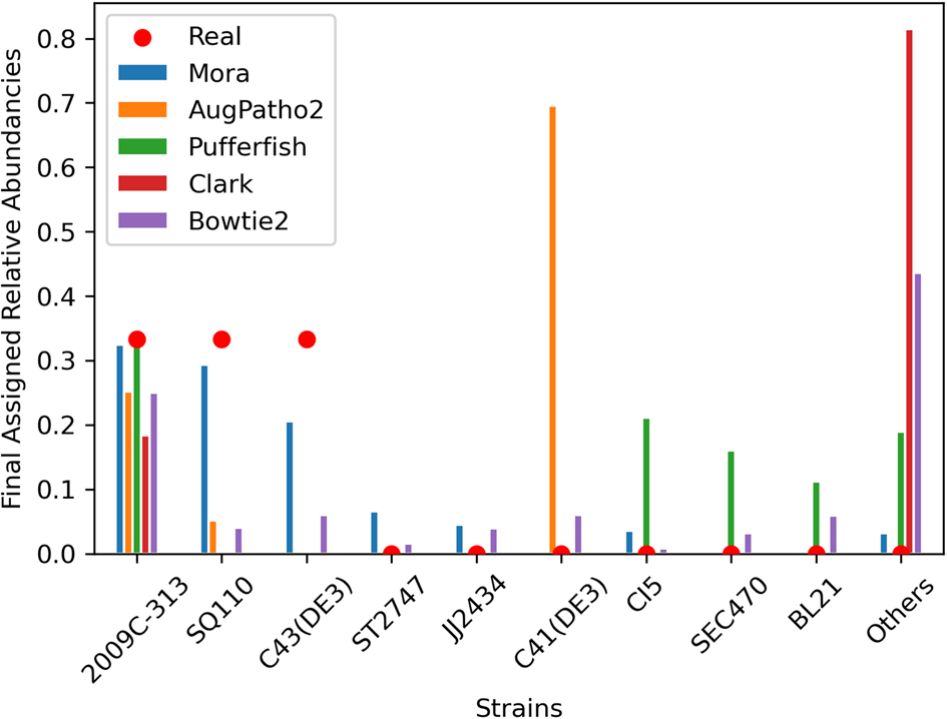
Relative assignment abundancies of Mora, AugPatho2, and Pufferfish of 950,000 synthetic short 150 bp pair-end reads to 58 *E. coli* strains. The synthetic short reads were simulated from the three *E. coli* strains: 2009C-3133, SQ110, and C43(DE3). The strains listed had at least 2000 assigned reads from at least one of the algorithms. Relative assignment percentages of the final assignments from three different algorithms are represented by the different coloured bars. The real abundance levels of the strains are represented by the red dots. AugPatho2 is Pathoscope2 but with slight changes in the code to make it able to run and output results. Assignment by Pufferfish and Bowtie2 were done by choosing the primary alignment in the SAM file, while assignment by Mora and AugPatho2 was done using Pufferfish as the initial aligner. Clark and Bowtie2 were run without any additional algorithms. Mora’s assignments are closest to the real abundances

To test strain identification, 58 *E. coli* reference genomes were obtained from NCBI and 950,000 short 150 bp pair-end reads were generated for 3 of those strains, so each strain has about 30x coverage. We aligned the reads with Pufferfish/Puffaligner [28], a very efficient read alignment method. We then used Mora and AugPatho2 to re-assign the reads, and we report the number of assigned reads for each strain. We also performed assignment using Clark and Bowtie2 with their default parameters. The results are shown in Fig. 3. AugPatho2 is programmed to perform an initial assignment by calling Bowtie2 with pathoMAP. To allow the use of other alignment algorithms, we generated the SAM file independently of AugPatho2 and ran pathoID and pathoMAP on it. Since all the strains had the same taxonomic ID, Kraken2 could not be benchmarked on this dataset. To run Clark, the taxonomic IDs were changed to be unique from each other.

In Fig. 3, Mora, AugPatho2, and Pufferfish were able to identify the presence of all three strains. Mora had a F1 score of 45.02, much higher than 29.01, 32.48, 18.35, 32.28, the F1 scores of AugPatho2, Pufferfish, Clark, and Bowtie2 respectively. AugPatho2 mapped most reads to the strain C41(DE3), a strain whose average nucleotide identity to C43(DE3) is 99.94% according to OrthoANI [29]. This may be caused by how Pathoscope2 incorporates reference length into its calculations of read alignment scores. The length of strain C43(DE3) was 56,061 base pairs shorter than strain C41(DE3), making the alignment scores to C41(DE3) higher than those to C43(DE3). As most high mapping scores were equal in value, selecting the primary alignment without regard of abundance levels is likely the reason for Pufferfish’s high false positive rate. Clark was unable to align 81% of the reads while Bowtie2 assigned almost equal amounts of reads to all strains. The number of reads assigned to each strain can be found in Additional file 1: appendix Table A.1.

### Large-scale simulated short read re-assignment at the species and genus level

We used the complex Illumina 400 dataset of 400 different microbial genomes and its corresponding simulated 26.6 million 75 bp paired-end short reads [30] to test the accuracy of short read alignment. We consider two cases: when there is a good guess of which genomes the reads come from, and when there is no information at all. For the first case, we built a reference library (REF-1) from the bacterial_data.txt file (478 genomes) that was used to simulate the reads. For the second case, we built a reference library (REF-2) using the complete bacterial genomes from NCBI RefSeq (6487 genomes). Importantly, REF-2 does not contain all references in REF-1. REF-2 contains 26% of the strains, 73% of the species, and 81% of the genera of REF-1. When using REF-2 as the reference genomes, scores for the strain rank are not reported due to the low number of strains from REF-1 included in REF-2.

When aligning to REF-1, Pufferfish was unable to map 12.7 million reads while Bowtie2 was unable to map 4.1 million. When aligning to REF-2, Pufferfish was unable to map 15.7 million reads while Bowtie2 was unable to map 9.7 million.

As shown in Tables 1 and 2, AugPatho2 performed only slightly better than Mora for both aligners, but the difference is within 3 points. However, it was much slower in runtime due to the pathoREP module in AugPatho2. For large data sets, the pathoREP module wasn’t able to output the final XML file due to lack of memory. We had to split the SAM file into 3–5 smaller files and change the XML output script to accommodate this problem. As seen in Table 3, Mora was over 19 times faster than AugPatho2, while only having a 3 times increase in RAM usage. Using Bowtie2 increased scores significantly due to its ability to map more reads than Pufferfish, but did result in a large increase in runtime. On REF-1, Clark had slightly better scores than Mora when using Bowtie2 at the species and genus level, but had low scores at the strain level. Kraken2 performed better than Mora for both aligners on REF-2 but was worse than Mora when using Bowtie2 at the species level on REF-1.

**Table 1:** Scores of algorithms when aligning short reads to REF-1

Algorithm	Strain			Species			Genus		
	F1	Sensitivity	Precision	F1	Sensitivity	Precision	F1	Sensitivity	Precision
Pufferfish	62.12	47.27	90.58	66.04	50.25	96.29	67.92	51.68	99.05
Mora + Pufferfish	66.20	50.37	96.53	67.04	51.01	97.75	68.49	52.11	99.87
AugPatho2 + Pufferfish	66.62	50.69	97.14	66.90	50.90	97.55	68.49	52.11	99.87
Bowtie2	84.54	78.04	92.20	88.94	82.11	97.01	91.21	84.21	99.48
Mora + Bowtie2	87.59	80.86	95.54	89.22	82.37	97.32	91.48	84.46	99.79
AugPatho2 + Bowtie2	89.03	82.19	97.10	89.64	82.76	97.77	91.50	84.47	99.80
Kraken2	NA	NA	NA	88.19	83.57	93.36	92.36	87.52	97.77
Clark	59.38	55.21	64.23	91.12	84.71	98.57	92.04	85.58	99.57

Both Mora and AugPatho2 were able to improve the scores of the initial aligner, though AugPatho2 appears to have a slight systematic edge

**Table 2:** Scores of algorithms when aligning short reads to REF-2

Algorithm	Species			Genus		
	F1	Sensitivity	Precision	F1	Sensitivity	Precision
Pufferfish	46.23	32.61	79.38	55.54	39.18	95.36
Mora + Pufferfish	47.79	33.72	82.06	56.00	39.50	96.15
AugPatho2 + Pufferfish	48.40	34.14	83.11	56.11	39.58	96.34
Bowtie2	64.08	52.36	82.58	74.96	61.24	96.59
Mora + Bowtie2	65.23	53.29	84.06	75.17	61.41	96.87
AugPatho2 + Bowtie2	66.10	54.01	85.18	75.27	61.24	96.99
Kraken2	70.28	66.32	74.73	88.54	83.55	94.15
Clark*	13.78	7.80	59.11	20.13	11.39	86.37

On both aligners, Mora performed just a bit worse than AugPatho2, with scores having a difference of at most 1 when using Bowtie2. Mora’s scores are consistently better than the aligners, showing that the re-assignment step is beneficial. Kraken2 was able to map more reads compared to Mora and AugPatho2 due to mapping more reads than Pufferfish and Bowtie2. Clark has an asterisk to signify that its low scores are due to an issue with building the bacteria database

**Table 3:** Wall clock time (s) and maximum RAM usage (GB) for algorithms on assigning simulated short reads (SR)

Algorithm	SR to REF-1		SR to REF-2	
	Time (s)	RAM (GB)	Time (s)	RAM (GB)
Mora	477.15	47.24	657.80	47.52
AugPatho2	28588.09	51.58	29278.85	15.99
Pufferfish	101.64	10.89	325.29	10.54
Bowtie2	726.44	2.91	894.84	26.85
Kraken2	73.48	2.40	70.54	24.33
Clark*	300.97	45.69	272.01	41.17

Other than using more RAM on short reads to REF-2, Mora consistently used fewer resources than AugPatho2 and was substantially faster. Time cannot be directly compared between the Mora/AugPatho2 and Kraken2/Clark as they require the output from one of the initial aligners. Clark has an asterisk to signify that had errors in building the REF-2 database, which resulted in less resources used. CPU time and other information can be found in Additional file 1: appendix Table A.4. All algorithms were run with 8 threads on a Google cloud virtual machine instance of type c2-standard-60

### Re-assignment of long reads

To test Mora on long reads, we used badreads [31] v0.4.0 with parameters for nanopore2020 reads at length 2500, standard deviation 1500 on REF-1 to simulate 203 thousand long nanopore reads. When using Minimap2 to perform the initial alignment to REF-1, 4 thousand reads could not be aligned. When using Minimap2 to align to REF-2, 30 thousand reads could not be aligned.

From Tables 4 and 5 we see that Mora’s scores on long reads for REF-1 are very similar to AugPatho2, and was slightly better when looking at the scores for REF-2. However, Table 6 shows that Mora performs at least four times faster and uses up to 10 fold less RAM compared to AugPatho2 on long reads. The main bottleneck for AugPatho2 was again PathoREP. Even after the augmentations of PathoREP to reduce its runtime and memory usage, AugPatho2 still had higher runtime and RAM usage than Mora for long reads.

**Table 4:** Scores between Minimap2, Mora, AugPatho2, Kraken2, and Clark when aligning long simulated reads to REF-1

Algorithm	Strain			Species			Genus		
	F1	Sensitivity	Precision	F1	Sensitivity	Precision	F1	Sensitivity	Precision
Minimap2	77.94	76.49	79.44	92.56	90.83	94.34	96.50	94.70	98.36
Mora	78.45	76.98	79.97	93.08	91.34	94.88	96.64	94.84	98.51
AugPatho2	78.95	78.87	79.02	94.26	94.17	94.35	97.68	97.01	97.78
Kraken2	NA	NA	NA	84.15	78.17	91.13	89.01	82.68	96.30
Clark	45.62	41.55	50.58	86.13	78.44	95.49	88.19	80.32	97.78

Both Mora and AugPatho2 increase the scores of Minimap2, but their scores are very similar

**Table 5:** Scores between Minimap2, Mora, AugPatho2, Kraken2, and Clark when aligning long simulated reads to REF-2

Algorithm	Species			Genus		
	F1	Sensitivity	Precision	F1	Sensitivity	Precision
Minimap2	65.65	60.32	72.01	83.74	76.95	91.86
Mora	68.86	63.26	75.55	83.96	77.13	92.11
AugPatho2	67.93	63.05	73.62	84.21	78.16	91.27
Kraken2	73.10	68.65	78.16	85.19	80.01	91.09
Clark*	22.97	14.83	50.98	36.10	23.30	80.12

Both Mora and AugPatho2 increase the scores of Minimap2. Mora slightly outperforms AugPatho2 at the species level but has slightly lower F1 and sensitivity scores at the genus level. Clark has an asterisk to signify that had an issue with building the database, which resulted in the low scores

**Table 6:** Wall clock time (s) and maximum RAM usage (GB) for algorithms on assigning simulated long reads (LR)

Algorithm	LR to REF-1		LR to REF-2	
	Time (s)	RAM (GB)	Time (s)	RAM (GB)
Mora	56.32	0.77	98.72	2.04
AugPatho2	341.93	21.52	429.23	22.522
Minimap2	1074.89	11.228	8696.29	20.048
Kraken2	193.08	2.58	326.09	25.01
Clark*	56.68	40.80	51.31	36.65

Mora used significantly fewer resources than AugPatho2 and was substantially faster. Time and RAM cannot be directly compared between the Mora/AugPatho2 and Kraken/Clark as Mora and AugPatho2 requires Minimap2 to give them an output file. Clark has an asterisk to signify that had errors in building the REF-2 database, which resulted in less resources used. All algorithms were run with 8 threads on a Google cloud virtual machine instance of type c2-standard-60

Kraken2 had lower F1 scores than Mora in REF-1, but had better scores in REF-2. Clark had low scores at the strain level for REF-1, while its scores for REF-2 were low due to an error in its database (see “Benchmarking details” section).

### Results on real sequencing data

To show applicability to real sequencing data, we ran Mora on two sets of real data, each representing a different scenario.

#### Disambiguating *E. coli* strains from real short reads

**Fig. 4:**
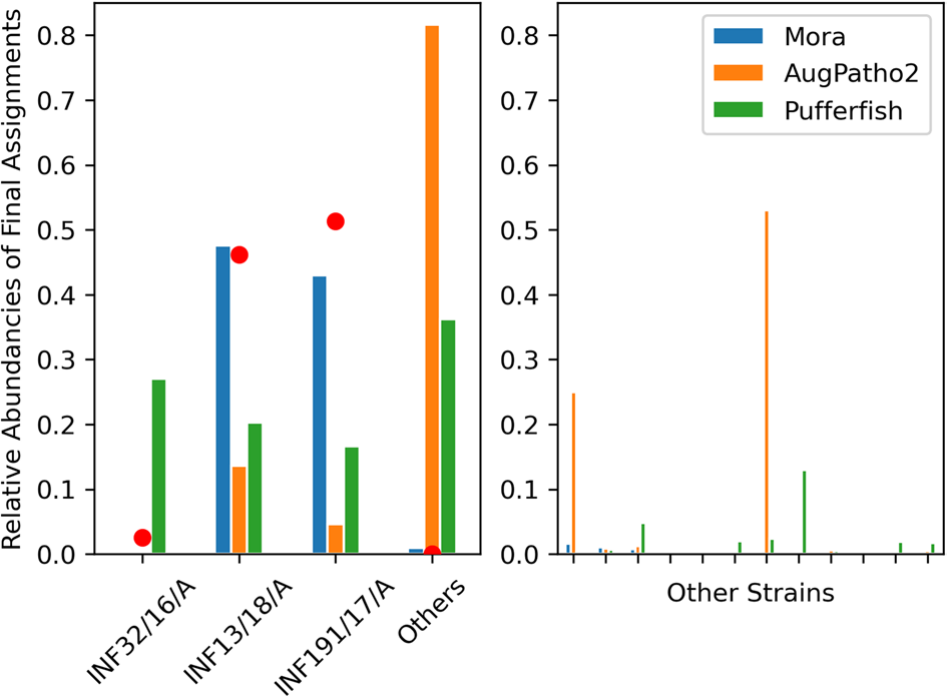
Comparison of assignment percentages of real short *E. coli* reads from the three assemblies: INF32/16/A, INF13/18/A, and INF191/17/A by Mora, AugPatho2, and the initial aligner Pufferfish. The real abundance levels of the strains are represented by the red dots. The left subfigure shows the real abundances as red dots, which are at 2.5% 46.18%, and 51.20% respectively. This highlights that Mora has the smallest $l^1$ error. Mora assigns 3% of reads to INF32/16/A while AugPatho2 assigns 1% of reads to it. The right subfigure shows 10 of the other strains that had the highest assignments from Mora. AugPatho2 and Pufferfish assigns a lot of reads to a wrong strain

In the first case, we look at real short *E. coli* reads from three different assemblies. Mora and AugPatho2 (with Pufferfish as the initial aligner) were run on 1481219 pair-end short reads of average length 250 bp. These reads were composed of reads from three SRA runs representing the assembly genomes of INF13/18/A, INF191/17/A, and INF32/16/A (see Additional file 1: Appendix Table A.7), while the references were the 58 *E. coli* strains used previously with the addition of the three new strains. The average nucleotide identity (ANI), calculated by skani [32], of the three strains to each other were between 96.4 and 96.57 (see Additional file 1: Appendix Table A.7). As seen in Fig. 4, Mora was able to assign more reads to the three INF strains compared to the other algorithms. The left subfigure shows that Mora has the smallest $l^1$ error, while the right subfigure shows that every other strain had very low read assignments from Mora. AugPatho2 assigned a lot of reads to a different complete genome assembly: JJ2434. The ANI between JJ2434 and INF191/17/A was 99.39, but its complete genome (i.e. one single contig) sequence length was 3.4 million bps longer than the longest scaffold in the assembly for INF191/17/A. This is the likely reason for the large error in AugPatho2, which uses reference lengths for scoring. Pufferfish assigns a lot of reads to the three INF strains equally, but also assigns a sizable amount of reads to another complete genome assembly: C41(DE3). Mora had an F1 score of 67.77 while AugPatho2 and Bowtie2 had F1 scores of 19.08 and 30.06 respectively.

#### Disambiguating real long-read community

**Fig. 5:**
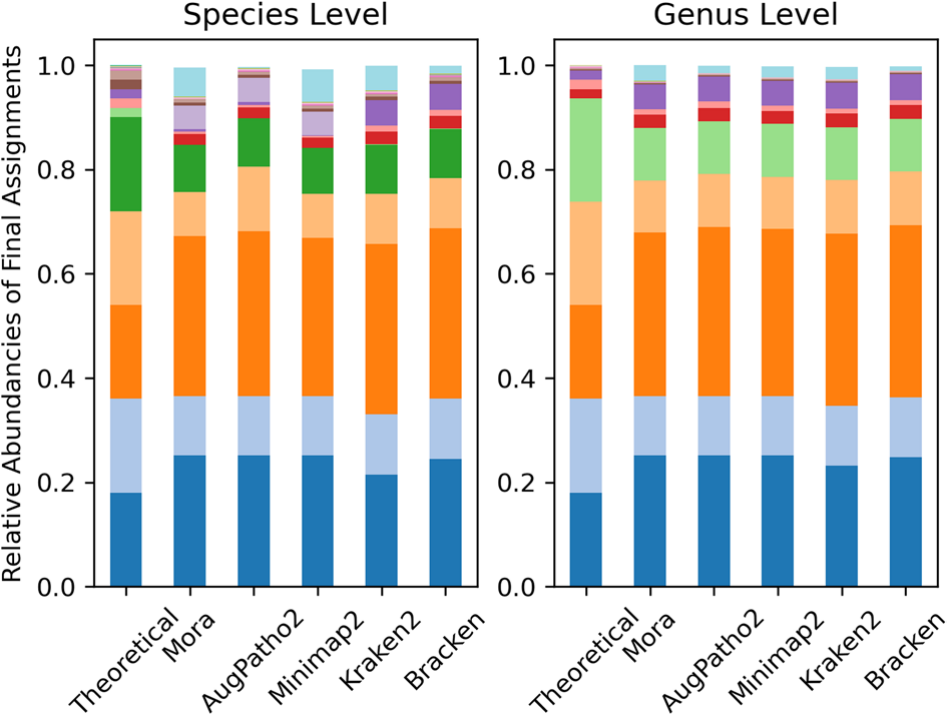
Species and genus abundance based on final read assignments of 5 different algorithms on the ATCC MSA-1003 mock community. Different coloured blocks in each column represent a different species/genus. The sub-figure on the left shows 24 of the most abundant species detected by the algorithms, with the light blue block at the top representing the other species. The sub-figure on the right shows the 18 genera that theoretically make up the mock community, with the blue block at the top of the columns representing the other genera. The initial aligner for Mora and AugPatho2 was Minimap2 after performing a pruning step of low quality reads with MAPQ score less than 5. Assignments for Minimap2 were done by picking the primary strain

In the second case, we test Mora on real long reads, but of different species (Fig. 5). The PacBio HiFi reads of medium length 8.3 kb are from the ATCC MSA-1003 mock community, comprised of 20 bacteria species in staggered abundances (5 at 18%, 0.18%, 0.018%, and 0.002% abundance levels), with two genera have two different species. The reference database is composed of the bacteria, archaea, fungi, viral, protozoa libraries from NCBI RefSeq. As theses were long reads, Minimap2 was used as the initial aligner for Mora and AugPatho2.

As we do not know the true identity of each read in the data, we compare the different algorithms by looking at if they were able to detect the presence of the 20 species which theoretically comprises the sample. A species or genus is detected if at least 0.01% of reads were assigned to it. Of the 20 species, Kraken2 and Bracken were able to detect 18 of them, while the rest were only able to detect 16. Minimap2 aligns no reads to *Bacillus cereus*, one of the 20 species, while Kraken2 aligns 2.3 thousand reads to it. However, they both aligned more than 49 thousand reads to *Bacillus pacificus*. This difference resulted in Mora and AugPatho2 not detecting *B. cereus* at all, while Kraken2 and Bracken do. This also explains another one of the species. At the genus level, Kraken2 and Bracken detected 16 of the 18 genera, while the other three algorithms only detected 15 of the 18 genera. The difference is most likely caused by the difference in alignments by Kraken2 and Minimap2.

As seen in Table 7, while Mora’s F1 score is low (primarily due to downstream effects from minimap2’s false positives), it still has a higher F1 score compared to Minimap2, so it is an improvement to just using the initial aligner. It is important to note that species detection is not the best use-case for Mora and that the F1 score is sensitive to the initial aligner’s mismappings. For $l^1$ scores, Mora’s $l^1$ error is 0.650, compared to Braken which has the lowest $l^1$ error of 0.568 at the species level. At the genus level, Mora’s $l^1$ error is 0.545 which is comparable to the the lowest $l^1$ error of 0.531 achieved by Bracken. The full results can be found in Table A.11 in the Additional file 1: appendix file.

**Table 7:** F1 scores for species and genus detection of ATCC MSA-1003 mock community by 5 different algorithms

Algorithm	Species			Genus		
	F1	Sensitivity	Precision	F1	Sensitivity	Precision
Minimap2	26.67	80.00	16.00	47.62	83.33	33.33
Mora	37.64	80.00	24.62	55.56	83.33	41.67
AugPatho2	41.02	80.00	27.59	58.82	83.33	45.45
Kraken2	55.38	90.00	40.00	65.31	88.90	51.61
Bracken	52.94	90.00	37.50	64.00	88.90	50.00

Kraken2 has the highest F1 score for both species and genus levels. Mora and AugPatho2 improve minimap2’s classifications, although their value is lower than Kraken2/Bracken

Mora’s runtime and memory usage is much lower than the other methods on this dataset. As seen in Table 8, Mora is more than 200 times faster than AugPatho2 while using around 10 times less RAM. Though these values are not comparable due to requiring alignment prior to usage, its ability to only require a SAM/BAM file can make it more modular and faster if the SAM/BAM file already exists.

**Table 8:** Wall clock time (s) and maximum RAM usage (GB) for algorithms on assigning long reads from ATCC MSA-1003 mock community

Algorithm	Time(s)	RAM(GB)
Mora	67.17	2.97
AugPatho2	15713.15	1.78
Minimap2	34712.47	23.87
Kraken2	522.15	76.79

Mora used fewer resources than AugPatho2 and was substantially faster. Time cannot be directly compared between the Mora/AugPatho2 and Kraken2 as they require the output from Minimap2. Bracken is not included as it is instantaneous once the Kraken2 result files are created. CPU time and other information can be found in Additional file 1: appendix Table A.4. All algorithms were run with 8 threads on a google c2-standard-60 machine type virtual machine

## Methods

We define *mapping score* as some positive value associated to each alignment that measures how good the alignment is. In practice, we use the AS:i secondary flag that is present in most read aligners, but our theory holds for any such score.

### Initial read mapping

The input to Mora is a SAM/BAM file with an initial alignment of the reads. After filtering out unaligned reads, the remaining reads are fed into Mora. At minimum, Mora only requires a SAM file with header information to output read (re)assignments. Mora optionally allows for the inclusion of taxonomic information of reference genomes in the final output by using accession and taxonomic information from NCBI.

For convenience, Mora is also implemented with a read mapping module using Snakemake [33], allowing the user to use Mora from a set of input reads and references. The mapping modules allow the user to use Pufferfish, Bowtie2, or Minimap2. However other programs can be used as long as their generated SAM contains a header with the reference genomes and the AS:i secondary mapping flag is available in each alignment record. After the SAM file is generated, the reads that could not be assigned are filtered out. These reads are later added to the final output with the “NO ALIGNMENT” string being assigned to them.

### Abundance estimation

We solve the problem of estimating the abundance levels of reference genomes by adapting the common model from calculating RNA transcript abundances used by Agamemnon [19] and RSEM [34]. In this model, each read is generated by first selecting a reference and then a position on that reference. Assuming the same model for all the reads, we have the following likelihood function for observing a set of reads from a set of reference genomes.

$$\begin{aligned} \mathfrak {L}(\theta |Q) = \prod _{r_i \in Q}{\sum _{j=1}^M{Pr(R_j|\theta )Pr(r_i|R_j)}} \end{aligned},$$

where M is the number of references, $Q$ is the set of reads, $\theta$ is the abundance estimation, $Pr(R_j|\theta )$ is the prior probability of selecting reference $R_j$, and $Pr(r_i|R_j)$ is the conditional probability of generating read $r_i$ from reference $R_j$.

$Pr(r_i|R_j)$ is computed by normalizing the mapping score between read $r_i$ and reference $R_j$ over all mapping scores from $r_i$. The estimation of $\theta$ is done with an EM algorithm. To reduce the number of iterations needed by the EM algorithm to converge, the reads are reduced to a set of equivalent classes where two reads $r_{i_1}$ and $r_{i_2}$ are equivalent if they align to the same set of references.

After every 10 iterations, we implement Agamemnon’s idea of using a set cover algorithm to remove redundant references with low abundances from the rest of the iteration process. This results in better-estimated abundances of leftover references while also increasing the convergence rate. These algorithms are implemented based on the Cedar algorithm used by Agamemnon. For more information, please consult Agamemnon’s original paper.

### Re-assignment of reads

Mora then adds to the functions of Agamemnon by using the estimated abundance levels to perform read re-assignment. The assignment of reads based on their mapping scores while trying to stay true to the estimated abundance levels can be modeled as a variant of the Weapon-Target Assignment (WTA) problem [35]. Given different weapons that are to be fired on a set of different targets, the objective is to find which weapons should be assigned to which targets to minimize the expected remaining health of the targets. Formulating it as a non-linear problem, let $\{T_1, T_2, \ldots , T_N\}$ be the set of targets and $\{W_1, W_2, \ldots , W_M\}$ be the set of different weapons. For each weapon type $W_i$, there are $w_i$ number of it and each has probability $p_{ij}$ to destroy the target $T_j$. After assigning $x_{ij}$ of weapon $W_i$ to target $T_j$, the probability that $T_j$ survives is $\prod _{i=1}^M(1-p_{ij})^{x_{ij}}$. Thus the WTA problem aims to minimize the following non-linear problem with constraints:

$$\begin{aligned} \min G(x) =& {} \min \bigg (\sum _{j=1}^N\prod _{i=1}^M(1-p_{ij})^{x_{ij}}\bigg ), \\ & {} \sum _{j=1}^Nx_{ij}\le w_i, \quad x_{ij}\in \mathbb {Z}^+. \end{aligned}$$

Instead of selecting which weapons to assign to which targets to maximize expected damage, we are selecting which reads to assign to which references to maximize likelihood. Let $R_j$ represent a reference genome, $r_i$ represent a read, and $p_{ij}$ represent the probability that $r_i$ comes from $R_j$ is true, calculated in the same way as for abundance estimation. To model our metagenomic problem as the WTA problem, we have three assumptions:

1. Every reference $R_j$ appears $a_j \cdot M$ times in the set of references, where $a_j$ is the estimated abundance of $R_j$ and *M* is the total number of reads. The value of $a_j \cdot M$ is approximated to the nearest integer.
2. Every read maps to some reference genome.
3. Every read is unique, so $w_i=1$ for $i=1, \ldots , M$.

These three assumptions result in the re-assignment problem being formulated as the following minimization problem with constraints:

1 $$\begin{aligned} \min F(x)= & {} \min \left( \sum _{j=1}^N\left[ \sum _{k=1}^{a_j\cdot M}\prod _{i=1}^M(1-p_{ij})^{x_{i,(j,k)}}\right] \right) \nonumber \\{} & {} \sum _{j=1}^N\sum _{k=1}^{a_j\cdot M}x_{i,(j,k)}\le 1, \quad x_{ij}\in \mathbb {Z}^+. \end{aligned}$$

The second sum shows that there are $a_j\cdot M$ of reference $M_j$ and the $x_{i, (j, k)}$ is how much of read $r_i$ we assign to the *k*th copy of reference $M_j$. This model is more punishing against undershooting compared to overshooting as over-assigning reads to a reference does not decrease the objective function *F*(*x*). This is desirable as it is better to identify all the low-abundance genomes and undershoot the most abundant genome than to not identify the low-abundance genomes. As leaving a read un-assigned and assigning it to a very wrong reference both contribute a value of 1 to the objective function *F*(*x*), the assumption that every read maps to some reference is needed to prevent large numbers of false positives. Using Eq. (1), we can now use optimization methods to get exact solutions, though this is not very practical given the bad runtime scaling for large data sets.

As this is an NP-hard problem [35], Mora uses a greedy algorithm that finds a relatively good solution. Mora views each reference as a bin with a fixed space capacity. Every time a read is assigned to a reference, the available capacity of the reference decreases. Once the capacity of a reference is full, no other read can be assigned to that reference unless something is taken out. The capacity $C_j$ of a reference $R_j$ is $C_j = a_j + 1/M$. As the amount of space each read takes up is 1/*M*, this implies that Mora at most over-assigns a single read to each reference. As reads get assigned to the references, the references ${R_i}$ can be represented as a list of assignments $\mathcal {A}(R_i)$ containing the reads that have already been assigned to it.

2 $$\begin{aligned} \mathcal {A}(R_i) = \{(r_{i_1}, s_{i_1, k}), (r_{i_2}, s_{i_2, k}),..., (r_{i_n}, s_{i_n, k})\} \end{aligned}$$

where $r_{i_k}$ represents a read, $s_{i_k, i}$ is the corresponding mapping score between $r_{i_k}$ and $R_i$, and *n* is the current number of reads that have been assigned to $R_i$. The list is ordered using $s_{i_k, i} \ge s_{i_{(k+1)}, i}$ and the capacity limitation is $\frac{n}{M} < C_i$. If $\frac{n+1}{M}\ge C_i$, we say that the reference $R_i$ is full. Similarly, the reads $r_j$ can be represented as a list of potential mappings $\mathcal {M}(r_j)$.

3 $$\begin{aligned} \mathcal {M}(r_j) = \{(R_{j_1}, s_{j, j_1}), (R_{j_2}, s_{j, j_2}),..., (R_{j_m}, s_{j, j_m})\} \end{aligned}$$

where the list is ordered such that $s_{j, j_k} \ge s_{j, j_{(k+1)}}$. The total score of a read $r_j$ is defined to be

$$\begin{aligned} T(r_i) = \sum _{(R_a, s_{i, a})\in \mathcal {M}(r_i)} s_{i, a} \end{aligned}$$

which gives us, by definition, that $p_{ij} = \frac{s_{i,j}}{T(r_i)}$.

Mora assigns the reads in terms of priority. A read $r_i$ is given priority 1 if the read maps to only one reference. A read $r_i$ is given priority 2 if the ratio of the second best score to the best score is less than a threshold. By default, this threshold is 0.5 but can be changed. A read is given priority 3 if it doesn’t satisfy the conditions of being priority 1 or priority 2. Once priority values are assigned, the priority 1 reads are assigned, followed by priority 2 reads, and then priority 3 reads.

Priority 1 reads are assigned to the unique reference they map to. For priority 2 reads, they are first sorted from highest best mapping score to lowest best mapping scores. In this order, the reads are assigned to the reference with the best mapping score if that reference has space. If the reference is at full capacity, the read is relabeled as a priority 3 read. When assigning priority 3 reads, all mappings between the priority 3 reads and references that still have space are sorted in terms of the score into a list. The reads are then assigned in order of this list, or left over for a second round of assignment if all of its potential references are full. After the initial assignment is done, Mora will try to “open up space” in a reference to assign leftover reads.

#### Definition 1

For a read $r_i$ and $(R_{j}, s_{i,j}) \in \mathcal {M}(r_i)$, $R_j$ can open up space for $r_i$ if it is full and there exists a $(r_{k}, s_{k,j})\in \mathcal {A}(R_j)$ such that $r_{k}$ can be moved to another reference $(R_l, s_{k,l})\in \mathcal {M}(r_k)$ with the condition that

4 $$\begin{aligned} \frac{s_{i,j}}{T(r_i)} - \frac{s_{k,j}}{T(r_k)} \ge \frac{s_{k,j}}{T(r_k)} - \frac{s_{k,l}}{T(r_k)}. \end{aligned}$$

Using the notation in this definition, we have the following theorem.

#### Theorem 1

If $r_i$ is a read and $R_j$ is a reference that can open up space for $r_i$ by re-assigning $r_k$ to another reference $R_l$, then doing so and then assigning $r_i$ to $R_j$ decreases the value of *F*(*x*) from Eq. (1).

#### Proof

The act of performing this re-assigning $r_k$ from $R_j$ to $R_l$ and then assigning $r_i$ to $R_j$ is equivalent to changing from $[x_{ij}, x_{kj}, x_{kl}] = [0, 1, 0]$ to $[x^*_{ij}, x^*_{kj}, x^*_{kl}] = [1, 0, 1]$. Since $p_{ij} = \frac{s_{i,j}}{T(r_i)}$, the last condition of being able to open up space gives us that

$$\begin{aligned} p_{ij} - p_{kj} \ge p_{kj} - p_{kl} \Longrightarrow (1-p_{ij}) + (1-p_{kl}) \le 2(1-p_{kj}) \le 1 + (1-p_{kj}). \end{aligned}$$

The right side can be written as

$$\begin{aligned} (1-p_{ij})^{x^*_{ij}} + (1-p_{kj})^{x^*_{kj}} + (1-p_{kl})^{x^*_{kl}} \le (1 - p_{ij})^{x_{ij}} + (1-p_{kj})^{x_{kj}} + (1-p_{kl})^{x_{kl}}. \end{aligned}$$

Since there is space in $R_l$, changing the three values $x_{ij}, x_{kj}, x_{kl}$ doesn’t result in the invalidation of any constraints and doesn’t affect any other terms in the sum of *F*(*x*). Thus, performing the re-assignment causes a decrease in the value of *F*(*x*). $\square$

If space cannot be opened up in $R_{j_1}$, Mora will try to open up space in $R_{j_2}$, and so on. If space cannot be opened for any of the references the read maps to, the read will be left to the end and be left un-assigned.A simple example of this greedy algorithm is shown in Fig. 2.

### Evaluation metrics

Genomes are classified as the same species or genus depending on the taxonomic information listed in NCBI. Taxonomic information of the data is obtained from NCBI Taxonomy’s FTP database (/pub/taxonomy/accession2taxid/) using the live and dead nucleotide sequence records. Genomes are classified as the same strain if their accession numbers are the same. The calculation of the accuracy metrics only considers reads that were successfully aligned by the first assignment algorithm. This allows us to evaluate the re-assignment algorithms without having the results be affected by the first assignment algorithms.

Read assignment accuracy on the simulated data sets is measured using F1 score, sensitivity, and precision. Let $r_i$ be a read generated from a reference $R_i$. At any taxonomic rank, $r_i$ is labeled as a true positive if it is mapped to reference $R_j$ that agrees with $R_i$ at that taxonomic rank. If $R_i$ and $R_j$ do not agree at that rank, then $r_i$ is labeled a false positive. If $r_i$ is not assigned to anything, it is not labeled as anything. For example, a read generated from *Escherichia coli* with accession number CP0001, assigning it to *Escherichia coli* with accession number CP0005 would be a true positive for the species and genus rank, but a false positive at the strain rank. Assigning it to *Escherichia fergusonii* with accession number CP1001 would be a true positive at the genus rank, but a false positive at the strain and species rank.

For a taxonomic rank, let TP be the total number of true positives for that rank and let FP be the total number of false positives for that rank. We define

$$\begin{aligned} \text {Sensitivity} = \frac{TP}{M}, \quad \text {Precision} = \frac{TP}{TP + FP}. \end{aligned}$$

where *M* is the total number of reads. The F1 score is defined to be the harmonic mean of sensitivity and precision. The $l^1$ score for final abundances is the sum of absolute value of the differences between the resulting abundance levels and the real abundance levels.

### Data simulation and availability

30 *E. coli* genomes assemblies were downloaded from NCBI Assembly and combined to form 58 *E. coli* strains. The three strains 2009C-3133, SQ110, and C43(DE3) were chosen randomly to simulate short reads 1.37 million 150 bp pair-end short reads. The reads were simulated according to a uniform distribution using art_illumina [36] with the default parameters for pair-end reads. The simulated 26.6 million 75 bp pair-end short read data from REF-1 was obtained from [30], where it was simulated using iMESS_Illumina with a skewed distribution. For the simulation of long reads, Badread [31] was used with the default parameters corresponding to mediocre Oxford Nanopore2020 reads with quantity 100 M. The read distribution is uniform and proportional to the length of the references.

The real E.coli data for the strains INF32/16/A, INF191/17/A, and INF13/18/A can be found at SRR15443628, SRR15497613, and SRR10587526 respectively. The ATCC MSA-1003 mock community dataset (PRJNA546278: SRX6095783) can be found on SRA.

For a use-case, the *E. coli* reads and references are available at https://github.com/AfZheng126/MORA-data. The full Additional file 1: appendix tables of scores, time, and memory usage for the different simulations are also available in the same repository as the *E. coli* reads/reference data.

## Conclusion

In this work, we presented Mora, a new flexible algorithm and pipeline for assigning reads at the strain level. Mora takes as input an alignment file and re-assigns the reads to strains by (1) estimating abundance information and (2) modelling the re-assignment problem as a discrete non-linear minimization problem for which Mora’s heuristic solution has provable guarantees. We showed that Mora performs well compared to other read assignment algorithms, but truly shines on reads from very similar strains.

Additionally, we showed that Mora is fast and practical to use, even on large datasets, with speeds and memory usage several times better than AugPatho2. Though the speed of the full pipeline is slower than Kraken2 and Clark and has lower scores at the species and genus level, it makes up for it this with higher F1 scores at the strain level on all types of reads, especially on reads from similar strains.

We found that there is a surprising lack of well-engineered tools that deal with the specific problem of sensitive read re-assignment to the strain level. Thus we designed Mora using general mathematical formulations, leading to it working well on many kinds of data. Furthermore, Mora is engineered to be modular and easy to use—the minimal input required is just a single SAM/BAM file. Thus we believe that Mora will be a useful tool for researchers interested in studying strain-level read information from metagenomic sequencing data.

### Supplementary Information

**Additional file 1.** GitHub repository containing all experimental results, the code for AugPatho2, and test data for running Mora. 

## Data Availability

All analysis software written for this manuscript are available in the https://github.com/AfZheng126/MORA repository, which contains the Mora software described in this manuscript. The software requires Rust >1.60.0 and has been tested on Linux environments. It is available under an MIT-style free and open source license. The datasets supporting the conclusions of this article are available in the https://github.com/AfZheng126/MORA-data repository. The code for AugPatho2 can also be found in the same repository.
